# On-target restoration of a split T cell-engaging antibody for precision immunotherapy

**DOI:** 10.1038/s41467-019-13196-0

**Published:** 2019-11-26

**Authors:** Agnes Banaszek, Thomas G. P. Bumm, Boris Nowotny, Maria Geis, Kim Jacob, Matthias Wölfl, Johannes Trebing, Kirstin Kucka, Dina Kouhestani, Tea Gogishvili, Bastian Krenz, Justina Lutz, Leo Rasche, Dirk Hönemann, Hannes Neuweiler, Julia C. Heiby, Ralf C. Bargou, Harald Wajant, Hermann Einsele, Gert Riethmüller, Gernot Stuhler

**Affiliations:** 1 University Clinic Würzburg, Department of Internal Medicine II, Hematology and Oncology, Würzburg, Germany; 2 University Clinic Würzburg, Children’s Hospital, Pediatric Oncology, Hematology and Stem Cell Transplantation, Würzburg, Germany; 3 University Clinic Würzburg, Department of Internal Medicine II, Division of Molecular Internal Medicine, Würzburg, Germany; 4 Department of Biotechnology and Biophysics, University Würzburg, Würzburg, Germany; 5 Comprehensive Cancer Center Mainfranken, Universitätsklinikum, Würzburg, Germany; 6 Ludwig-Maximilians-University, Institute for Immunology, Munich, Germany

**Keywords:** Biotechnology, Tumour immunology, Tumour immunology

## Abstract

T cell-engaging immunotherapies are changing the landscape of current cancer care. However, suitable target antigens are scarce, restricting these strategies to very few tumor types. Here, we report on a T cell-engaging antibody derivative that comes in two complementary halves and addresses antigen combinations instead of single molecules. Each half, now coined hemibody, contains an antigen-specific single-chain variable fragment (scFv) fused to either the variable light (V_L_) or variable heavy (V_H_) chain domain of an anti-CD3 antibody. When the two hemibodies simultaneously bind their respective antigens on a single cell, they align and reconstitute the original CD3-binding site to engage T cells. Employing preclinical models for aggressive leukemia and breast cancer, we show that by the combinatorial nature of this approach, T lymphocytes exclusively eliminate dual antigen-positive cells while sparing single positive bystanders. This allows for precision targeting of cancers not amenable to current immunotherapies.

## Abstract (web summary)

The restriction of appropriate tumour-specific antigens is a current limitation for T cell-engaging immunotherapy. Here, the authors have designed a new system constituted by two halve antibodies, which engage T cells once binding to two different antigens, to specifically eliminate double positive cells in preclinical leukemia and breast cancer mouse models.

## Introduction

Monoclonal antibodies against cancer represent one of the fastest growing fields in modern drug therapy. Among the many hundred therapeutic antibodies and antibody derivatives presently listed in preclinical and clinical studies, a few stand out which focus on retargeting cytotoxic T lymphocytes at malignant cells^1,2^. Of these, the most advanced are chimeric antigen receptors (CARs) transfected into T cells and bispecific T cell-engaging antibodies (BiTEs), both using a monospecific single-chain variable fragment (scFv) as a targeting device. By and large, the target molecules addressed by these antibody derivatives are differentiation antigens present on malignant cells as well as on their non-transformed counterparts, whose engagement often entails serious, if not lethal adverse events^3,4^.

As true tumor-specific antigens suitable for antibody-based therapeutics are rare, we here investigate a combinatorial approach that addresses antigen combinations aberrantly and uniquely expressed by certain types of leukemia or lymphomas^5^, or solid cancer and cancer stem cells of other provenance^6–10^. Furthermore and in light of the clinical efficacy of T cell-engaging therapies, we strive to redirect T lymphocytes for the lysis of tumor cells in a dual antigen-restricted fashion^11,12^.

To this end, we designed a tri-specific antibody split into two parts. Each of these halves, referred to as hemibody, is composed of an antigen-binding single-chain variable fragment (scFv1 or scFv2) fused to either the variable heavy (V_H_) or the variable light chain (V_L_) domain of a T cell-activating anti-CD3 antibody^13–16^. When a complementary pair of hemibodies binds to the respective antigens on the surface of a single target cell, the V_H_ and V_L_ domains align, re-associate, and reconstitute the original CD3-binding site. This way, CD3-positive T lymphocytes become activated and retargeted for tumor cell lysis.

## Results

### Antigen guided anti-CD3-Fv reconstitution

**Fig. 1:**
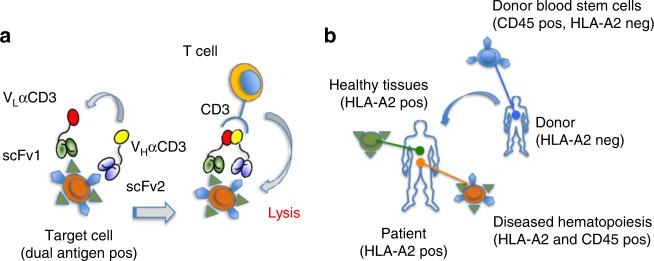
Combinatorial immunotherapy by hemibodies. **a** Binding of two hemibodies to their respective antigens on a target cell, each consisting of a single-chain variable fragment (scFv) fused to the variable light (V_L_) or variable heavy chain domain (V_H_) of a CD3-specific antibody, enables V_H_/V_L_ association and the reconstitution of a functional CD3-binding site to engage T cells. **b** Allogeneic mismatch transplantation model: The hematopoietic system of a HLA-A2-positive leukemia patient is replaced by blood stem cells of an HLA-A2-negative donor. Hemibodies directed against HLA-A2 and the pan-hematopoietic marker CD45 tag the patient’s dual antigen-positive diseased hematopoiesis for lysis by T cells, while sparing single antigen-positive healthy tissues and donor-derived blood cells.

Experimental evidence for the reconstitution of a CD3-binding site on-target (Fig. 1a) is provided by a clinically highly relevant in vitro model of allogeneic HLA-mismatch transplantation^17^, where blood stem cells from an HLA-A2-negative healthy donor replace the diseased hematopoietic system of an HLA-A2-positive leukemia patient (Fig. 1b). In this scenario, all nucleated cells of the patient express the selected HLA-A2 antigens, while the pan-hematopoietic lineage marker CD45 labels hematopoietic cells of recipient and donor origin alike. The only cells that co-express HLA-A2 and CD45 are hematopoietic cells of the patient including all malignant phenotypes.

**Fig. 2:**
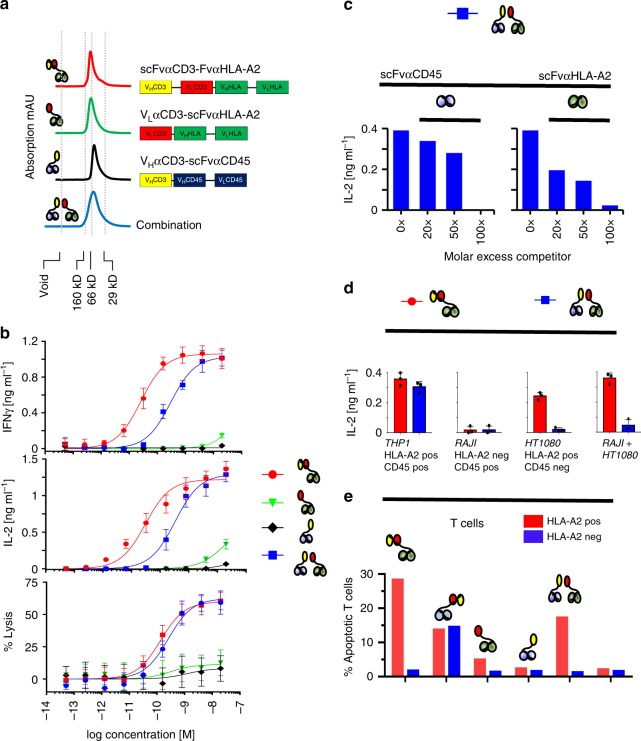
Dual antigen-restricted reconstitution of a functional CD3-binding site. **a** V_L_αCD3-scFvαHLA-A2 (green curve) and V_H_αCD3-scFvαCD45 (black curve) hemibodies were isolated by immobilized metal ion affinity chromatography (IMAC) and size-exclusion chromatography (SEC) and analyzed by FPLC at 1 mg/ml. V0 void volume/aggregates. Typical elution profiles of the hemibodies, the bispecific BiTE construct (scFvαCD3-FvαHLA-A2, red curve on top), and a mixture of the two hemibodies (blue curve, bottom) are shown for comparison. **b** HLA-A2/CD45 dual-positive THP-1 acute myeloid leukemia cells were co-incubated with HLA-A2-negative donor peripheral blood mononuclear cells (PBMCs) and constructs as indicated. T cell engagement was assessed by interferon-γ (IFN-γ) and interleukin-2 (IL-2) secretion and target cell lysis. The bispecific HLA-A2 × CD3 BiTE was used as a positive control. Data represent the mean values with standard deviations (±SD) from triplicate wells from three independent experiments; the effector to target cell ratio (E:T) was 5:1. **c** Binding of hemibodies (6 nM each) to THP-1 cells was competitively blocked by molar excess of scFv specific for CD45 (left) or HLA-A2 (right). Stimulation of donor PBMCs was assessed by IL-2 secretion at E:T = 2:1. Data represent the mean from two replicate wells from three experiments that yielded similar results. **d** Dual antigen-positive THP-1 leukemia, single-positve RAJI Burkitt lymphoma, or HT1080 fibrosarcoma cells were loaded with the hemibody pair (blue bars) or BiTEs (red bars), washed twice, and incubated with donor PBMCs as indicated at a E:T of 2:1. Secretion of IL-2 was assessed by ELISA. Data represent the mean (±SD) from two replicate wells from three experiments. **e** HLA-A2-positive (red bars) and HLA-A2-negative (blue bars) PBMNC were incubated with constructs as indicated (10 nM each). % apoptosis was assessed after annexinV staining by flow cytometry techniques as a measure of fratricide reaction. Data are representative for three independent experiments yielding comparable results.

As shown in Fig. 2a, we produced a hemibody pair addressing HLA-A2 (V_L_αCD3-scFvαHLA-A2; green) and CD45 (V_H_αCD3-scFvαCD45; black) as well as an HLA-A2 directed bispecific antibody of the BiTE class as control (scFvαCD3-scFvαHLA-A2; red) and analyzed the elution profiles of the constructs after size-exclusion chromatography (SEC). We found hemibodies and BiTEs to elute essentially as monomers. Moreover, hemibodies even when mixed prior to SEC did not hetero-dimerize or multimerize (Fig. 2a, bottom, blue curve), an observation that is important in light of exposed hydrophobic patches at the αCD3 V_H_ and V_L_ interface that in some experimental models leads to aggregation^13–16,18–20^. The overall design of the constructs are depicted in Fig. 2a and details on their architecture, purity, production yield, and long-term stability are provided in Supplementary Figs. 1 and 2.

### Restricted T cell activation to dual antigen-positive cells

To test whether hemibodies are able to form a CD3-binding site capable to trigger T lymphocyte effector functions, we first established an in vitro assay using HLA-A2 and CD45 dual antigen-positive THP-1 myeloid leukemic blasts and CD45-positive but HLA-A2-negative donor T cells. As presented in Fig. 2b, we found the combination of the two hemibodies to stimulate donor T cells to secrete interferon-γ (IFN-γ), interleukin-2 (IL-2), and to lyse tumor cells at half-maximal effective concentrations (EC_50_) of 290, 340, and 260 pM, respectively. In contrast, individual hemibodies neither stimulated cytokine release nor induced cytolytic activity in T cells when added separately from the other, except for the highest concentrations tested. For cytokine induction, the required hemibody concentrations are about 10 times higher as for the cognate BiTE molecule (26, 30, and 130 pM), which was deployed as a positive control. The higher activity of the BiTEs might be explained by their intact CD3-binding sites for effective T cell engagement. Hemibodies, on the other hand, first have to associate with the cognate partner in order to reconstitute a functional CD3-binding module.

To better understand anti-CD3-Fv reconstitution, we next asked whether the two hemibodies first have to bind to their respective target antigens on the tumor cell’s surface to trigger T cell function. To this end, we blocked the antigenic epitopes on CD45 or HLA-A2 on THP-1 cells using site-specific scFv competitors and tested the ability of the hemibody pair to induce IL-2 production by donor T lymphocytes. As demonstrated in Fig. 2c, both scFv competitors were able to completely inhibit T cell activation, indicating that both hemibodies have to bind to their target antigens in order to reconstitute the T cell triggering anti-CD3-binding site. Self-assembly of a functional CD3-binding unit after binding of only one hemibody, followed by side-to-side association of its partner independently of binding to the same cell surface, was therefore ruled out.

In line with these results, cell lines that have been loaded with both hemibody constructs but express only one of the two target molecules such as CD45 single-positive RAJI or HLA-A2 single-positive HT1080 cells did not induce IL-2 production by donor T cells (Fig. 2d, blue bars). Moreover, in this assay system, T cells were not stimulated in situations where the two target molecules were expressed on the surfaces of two different target cells (RAJI + HT1080). In contrast, a BiTE specific for HLA-A2 triggered T cells in the presence of double antigen-positive THP-1 cells, HLA-A2 single-positive HT1080 cells, and in situations where HT1080 were mixed with CD45 single-positive RAJIs (Fig. 1f, red bars). Thus, hemibodies but not BiTEs discriminate co-expression of two target molecules on a single cell basis.

Together, these data suggest that functional V-fragment complementation strictly depends on prior binding of both hemibodies to their respective target antigens on the same cell surface for productive T cell activation to occur. Moreover, BiTEs directed against antigens expressed on tumor and effector T cells, such as CD45 or HLA-A2 if HLA-A2-positive T cells are interrogated, induced massive fratricide reactions, indicating that co-expression of potential candidate target antigens on T lymphocytes may further limit the therapeutic scope of BiTE constructs (Fig. 2e).

### Antigen-dependent VH/VL complementation

To investigate the biochemical basis for conditional V-fragment complementation of the hemibody constructs, we next analyzed the inter- and intramolecular association events of the respective V_H_ and V_L_ domains involved. V_H_/V_L_ interactions are generally of low affinity and isolated V_H_ and V_L_ domains rapidly dissociate from each other at low concentrations and in the absence of suitable linkers^13–16,18^. However, polypeptides composed of two or more V-domains chained up in a row may dimerize due to multiple intermolecular V_H_/V_L_ interactions, a finding that was exploited for the generation of diabodies^21^.

**Fig. 3:**
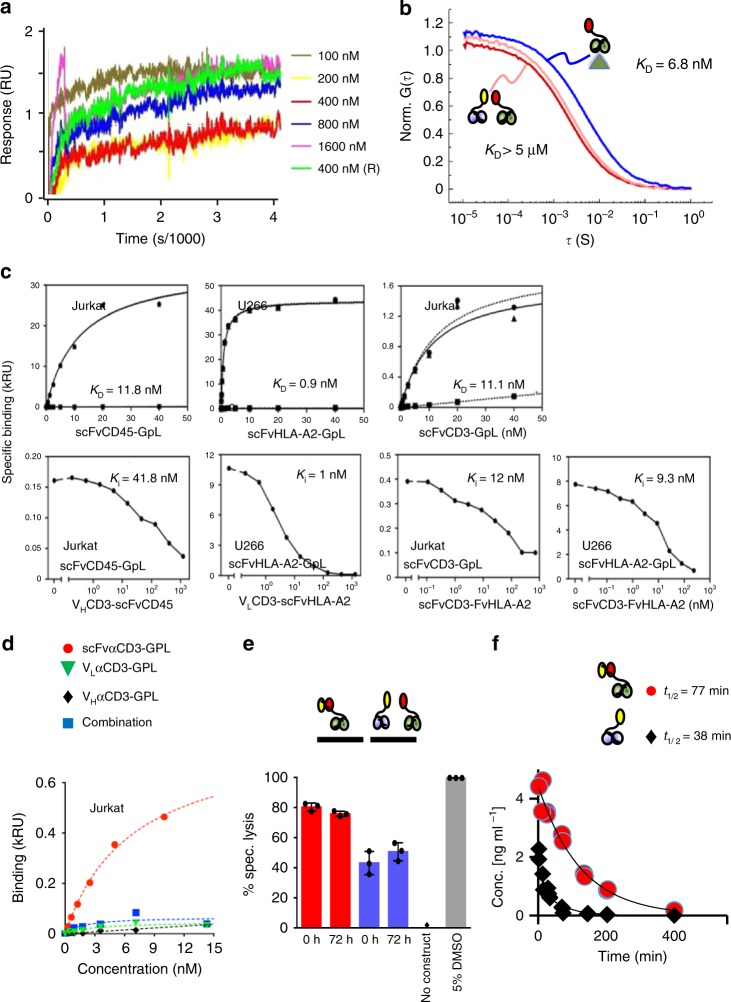
Biochemical basis for hemibody complementation. **a** A plasmon resonance chip was coated with V_L_αCD3-scFvαHLA-A2. V_H_αCD3-scFvαCD45 was used as an analyte at 100, 200, 400, 800, 1600 (nM) as indicated. Association contact time was 300 s, dissociation contact time 3.600 s at a flow rate of 30 μl/min, data represent one out of three experiments. **b** FCS autocorrelation functions, normalized to the number of molecules, recorded for 1 nM V_L_αCD3-scFvαHLA-A2 alone (dark red) and for the same sample in presence of 1 µM V_H_αCD3-scFvαCD45 (light red) or 100 nM HLA-A2 antigen (blue). Curves are fitted using data derived from three independent experiments **c** Top: Equilibrium binding study of scFv-GpL (*Gaussia princeps* luciferase) fusion proteins specific for CD45, HLA-A2, or CD3 to the respective antigens on 10^5^ Jurkat (CD3^+^, CD45^+^) and U266 cells (HLA-A2^+^, CD45^+^). Specific binding (triangles, solid line) was calculated as the difference of total (circles, dashed line) and non-specific binding (squares, dashed line) determined by using an irrelevant scFv-GpL fusion protein, HLA-A2-negative KMS-12-BM cells or CD3-negative U266 cells as indicated. Bottom: For heterologous competition analysis, cells were incubated with scFvCD45-GpL (2 nM), scFvHLA-A2-GpL (2 nM), or scFvCD3-GpL (4 nM) and the indicated concentrations of the hemibodies or the bispecific BiTE construct. IC_50_ values were determined and used to calculate the *K*_*i*_ of CD45-, HLA-A2-, and CD3-specific scFv domains by help of the previously determined *K*_D_-values of the scFv-GpL fusion proteins. Data show one out of three experiments yielding comparable results. **d** scFvCD3 or isolated V_H_CD3 and V_L_CD3 domains were fused to GpL. No specific binding of individual or combined constructs to CD3-positive Jurkat cells were detected as assessed by cell-bound luciferase activity. Data show one out of three experiments yielding comparable results. **e** Preservation of T cell triggering activity after incubation of hemibodies and BiTEs in human serum for 72 h at 37 °C. Read out was specific lysis at E:T of 5:1. Data represent the mean (±SD) from three replicate wells from one out of three experiments yielding comparable results. **f** In vivo serum half-lives after intravenous injection of 8 µg of a hemibody or BiTE construct into Balb/c mice. Values are deduced from >40 mice as determined by ELISA.

To test whether hemibodies hetero-dimerize via intermolecular V_H_/V_L_ interactions, we measured binding of the hemibody constructs using plasmon resonance. As shown in Fig. 3a, we could not detect any association signal within the sensitivity limit of the assay which is in the range of K_D_ = 10^−5^ M. To exclude artifacts from surface immobilization on the plasmon resonance chip, we next characterized the hemibody interaction directly in solution using fluorescence correlation spectroscopy (FCS). FCS analyzes the temporal behavior of fluorescence signals arising from molecules diffusing through the detection volume of a confocal microscope^22^. The FCS autocorrelation function decays at the characteristic time constant of molecular diffusion. This time constant increases with the molecular weight of the probed species and is thus a measure for hemibody dimerization. Employing FCS, we found negligible binding of hemibody V_L_αCD3-scFvαHLA-A2 to its V_H_αCD3-scFvαCD45 partner at µM concentration (K_D_ > ~ 5 µM), while we observed high-affinity binding of V_L_αCD3-scFvαHLA-A2 to its antigen HLA-A2 (K_D_ = 6.8 ± 0.6 nM) (Fig. 3b, Supplementary Fig. 3). These findings not only confirm results from plasmon resonance experiments but also indicate that hemibodies do not dimerize in solution, which can be explained by the overall design of the hemibodies and the order of the V-domains positioned in V_H_–V_H_–V_L_ and V_L_–V_H_–V_L_ orientation (Fig. 2a, Supplementary Fig. 1).

To impede intramolecular mispairing, we used short linkers to join the anti-CD3 V_H_ and V_L_ domains to the N-terminus of the antigen-specific CD45 and HLA-A2 scFvs. This way, the assembly of anti-CD3 V_L_ with the adjacent V_H_ domain in the HLA-A2 specific hemibody construct or the anti-CD3 V_H_ with the subsequent anti-CD45 V_H_ domain should be sterically hindered, thus facilitating correct folding of the scFv targeting moiety (Fig. 2a, Supplementary Fig. 1). To test this hypothesis, we first fused the anti-CD45, -HLA-A2 and -CD3 scFvs to *Gaussia princeps* luciferase (GpL) and determined dissociation constants (*K*_D_) of 11.8, 0.9, and 11.1 nM for the respective antigens in equilibrium binding studies on intact cells (Fig. 3c, upper panel). Heterologous competition analyses with the hemibodies and the bispecific BiTE control against the cognate GpL-scFv fusion proteins not only confirmed the affinities determined in the equilibrium binding studies but also proved the integrity of the CD45-, HLA-A2-, and CD3-specific paratops within the framework of the hemibody format, indicating correct intramolecular folding (Fig. 3c, lower panel).

Employing this highly sensitive read-out system, we next analyzed binding of luciferase-tagged anti-CD3 V_H_ and V_L_ variable domains (V_H_αCD3-GpL and V_L_αCD3-GpL) to the CD3 antigen on Jurkat cells. As shown in Fig. 3d, we detected negligible binding of either construct individually or in combination to CD3 as compared to the intact scFv αCD3-GpL fusion protein. These observations are in line with structural data indicating that both, V_H_ and V_L_ contribute to CD3 binding and suggest that for the anti-CD3 V-domains used in this study, even the ternary complex with the CD3 antigen is of low avidity^16,23,24^. Together, the biochemical data reported here provide the molecular explanation for dual antigen-restricted V-fragment complementation.

### Pharmacokinetics

In preparation for in vivo studies, we tested the stability of BiTEs and hemibodies in fresh human serum and detected steady T cell stimulating activity even after 72 h incubation at 37 °C, excluding significant degradation of the constructs by serum proteases (Fig. 3e). We determined the terminal half-lives (*t*_1/2_) of the constructs in vivo in mice after intravenous (i.v.) injection and found *t*_1/2_ = 38 min for the hemibody and *t*_1/2_ = 77 min for the BiTE molecule (Fig. 3f). These rather short half-lives are advantageous in early clinical studies because off-target toxicities can immediately be terminated by cessation of the drugs. For the anticipated in vivo mouse models, however, we resorted to the *subcutaneous* (s.c.) route of administration since s.c. injections of the bispecific peptides resulted in long-standing plasma concentrations with peak activities after 4–8 h.

### Hemibodies eliminate established tumors in vivo

**Fig. 4:**
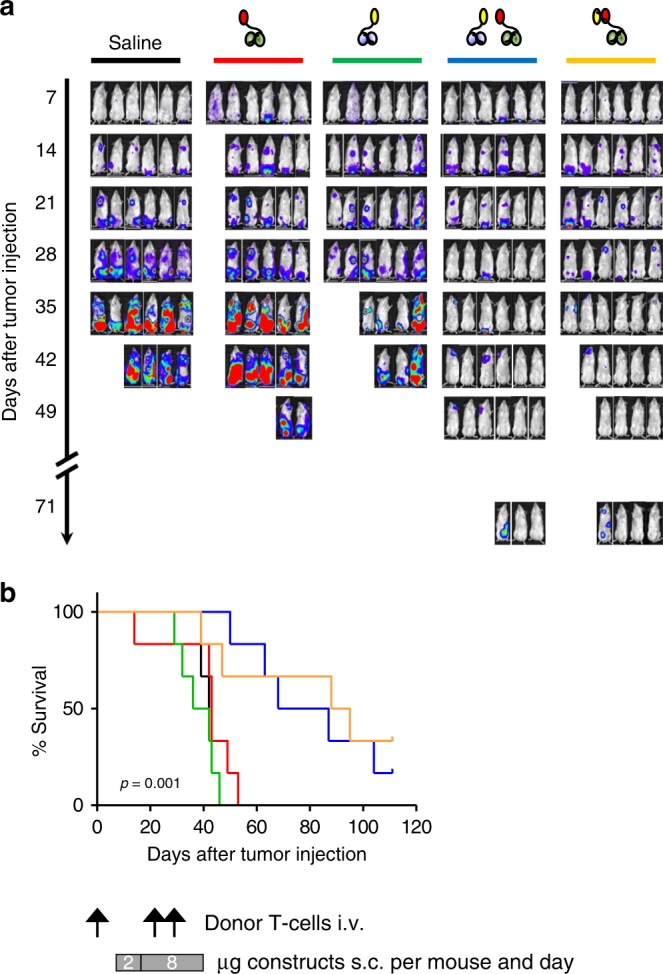
High precision cancer cell targeting in vivo. Immune deficient mice (6 per group) were challenged with 1 × 10^6^ luciferase-positive THP-1 (CD45 and HLA-A2 positive) tumor cells intravenously (i.v.) on day 1. HLA-A2 negative memory CD4 and CD8 donor T lymphocytes were added i.v. on day 1, 22, and 28. After tumor engraftment on day 7, mice were treated subcutaneously with either saline (PBS), individual hemibodies addressing CD45 or HLA-A2 antigens, the combination of both hemibodies, or the HLA-A2 targeting BiTE control with a starting dose of 2 µg/mouse per day for 1 week, followed by 8 µg/mouse per day at distant sites until day 39. **a** Tumor burden of luciferase-positive THP-1 cells were assessed on a weekly basis by IVIS Lumina XR Real-Time Bioluminescence Imaging and **b** survival was monitored daily until day 110. Significance was determined by the Kaplan–Meier estimator; *p* = 0.0017 for the hemibody combination versus V_L_αCD3-scFvαHLA-A2, *p* = 0.0005 for the hemibody combination versus V_H_αCD3-scFvαCD45. No significant difference for the hemibody combination versus the bispecific BiTE control, *p* = 0.6670. The data represent one experiment, statistical considerations on effect and group size are provided in Supplementary Table 1.

To put the potential therapeutic applicability of hemibodies to the test, immune deficient NOD/SCID gamma (NSG) mice were challenged i.v. with luciferase-labeled THP-1 tumor cells at day 1. T lymphocytes from a healthy donor were added i.v. at day 1, 22, and 28 (Fig. 4). After engraftment of tumor cells at day 7, saline, individual hemibodies, the combination of the two hemibodies or a BiTE control were injected s.c. daily until day 39. To investigate whether the hemibodies can find each other for functional complementation on-target, the constructs were injected separately from each other at distant sites, one in the neck, the other one in the thigh. While all mice receiving saline or single hemibodies rapidly developed progressive disease and met criteria for euthanasia within 53 days, mice treated with the hemibody pair or the BiTE control rejected established tumors (Fig. 4a). Interestingly, after discontinuation of the daily injections, some tumors in both cohorts recurred. This finding was not unexpected in light of the clinical experience with blinatumomab and the well-known need for long treatment periods for definite disease control^11^. Yet, overall survival was significantly prolonged in mice receiving the hemibody pair or the BiTE control (Fig. 4b).

### Bystander toxicity

**Fig. 5:**
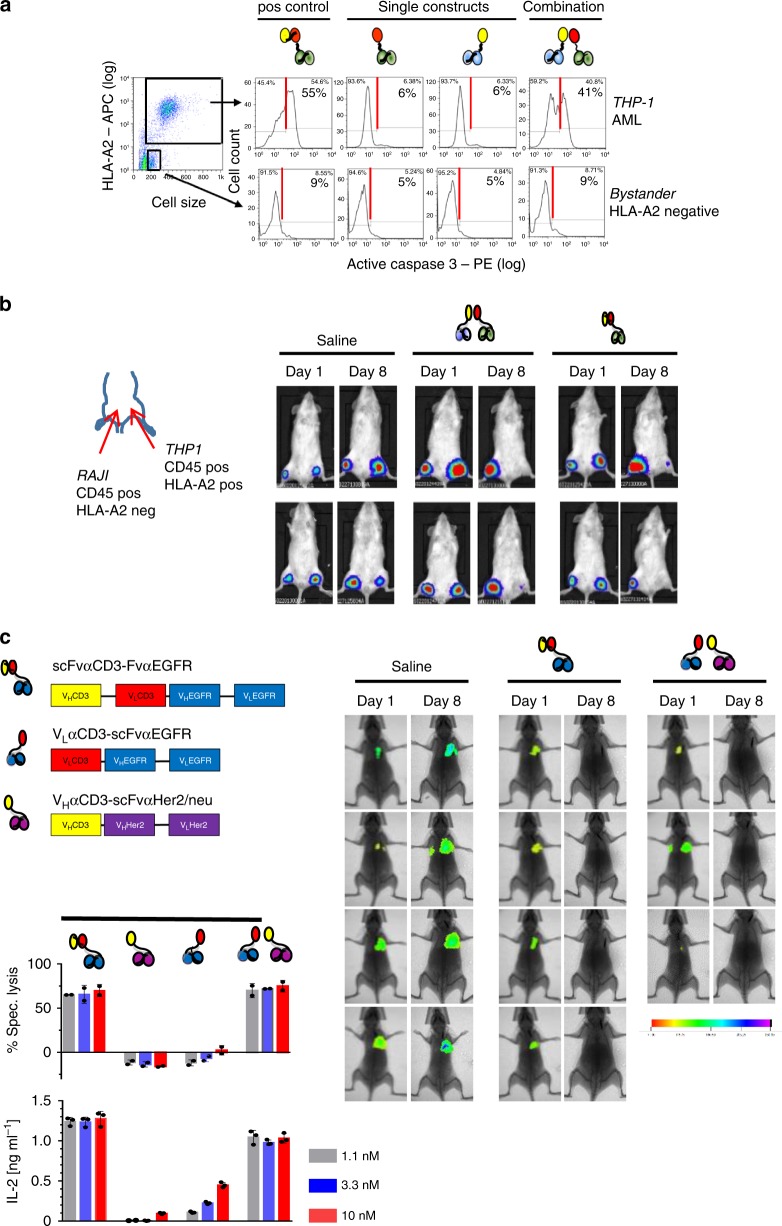
Absence of bystander cytotoxicity and translation into solid tumor model. **a** Intracellular caspase-3 activation as a measure of apoptosis in HLA-A2/CD45 dual-positive THP-1 (upper panels) and CD45-positive but HLA-A2-negative bystander cells (bottom panels) was assessed by flow cytometry in vitro after co-culture with donor PBMCs and CD45 or HLA-A2 targeting hemibodies as indicated (3 nM). The HLA-A2 specific BiTE was used as a positive control (left panel); data represent one out of two experiments that yielded similar results. **b** Luciferase-expressing dual antigen-positive THP-1 and single antigen-positive RAJI cells were injected subcutaneously in the left and right thigh, respectively. Donor T cells were injected i.v. at day 1 and saline (PBS), paired hemibodies and the BiTE control (8 µg/mouse) were administered s.c. daily until day 7. Data are representative for two independent experiments. **c** Immune deficient mice were challenged i.v. with luciferase-expressing human breast cancer cells MDA-MB-231, which co-express EGFR and Her2/neu antigens. After engraftment of lung metastases on day 3, PBMCs from a healthy donor were administered i.v. followed by s.c. injection of a buffer control (saline), a CD3 × EGFR BiTE or the combination of EGFR and Her2/neu-specific hemibodies. Tumor burden was visualized by IVIS Lumina XR Real-Time Bioluminescence Imaging on days 1 and 8. One in vivo experiment out of two is shown. For in vitro analyses, data represent means (±SD) of triplicate wells from at least two independent experiments, E:T = 10:1.

To investigate possible off-target effects, we assessed hemibody-mediated induction of apoptosis in dual antigen-positive targets and single antigen-positive bystander cells by measuring active caspase-3 on a single cell level. In the presence of donor T lymphocytes, we observed activation of intracellular caspase-3 exclusively in dual antigen-positive THP-1 tumor cells probed with the hemibody pair or the bispecific BiTE control. THP-1 cells exposed to each of the hemibodies separately remained unaffected (Fig. 5a, upper panel). In stark contrast, single antigen-positive monocytes treated in the same test tube only presented background levels of caspase-3 activation in either experimental condition, indicating that T cells are triggered exclusively on target, excluding substantial bystander toxicity in vitro (Fig. 5a, bottom).

To test adverse bystander toxicity in vivo, we next challenged NSG mice s.c. with luciferase-tagged dual antigen-positive THP-1 and single antigen-positive RAJI cells which in this setup represent bystander non-target tissues. After engraftment, donor T cells were administered i.v. at day 1, followed by daily s.c. injections of saline, the hemibody pair or the BiTE control for 1 week (Fig. 5b). We observed rejection of the THP-1 but not of RAJI cells by the hemibodies, indicating exquisite specificity and high precision targeting of dual antigen-positive target populations exclusively. The HLA-A2 directed BiTE served as a positive control.

### Hemibodies are active against breast cancer

To venture into solid tumor therapy, we targeted the combinatorial approach to Erb-b2 receptor tyrosine kinase 2 (Her2/neu) and epidermal growth factor receptor (EGFR) antigens. Both antigens are strongly associated with malignant diseases and are addressed by numerous state of the art antibody therapies. The expression of EGFR is closely associated with cell proliferation^25^, while Her2/neu is frequently upregulated on breast, ovarian, and other cancer cells^26^.

As shown in Fig. 5c, we engineered EGFR and Her2/neu reactive hemibodies and an EGFR-specific BiTE control and tested their abilities to redirect T cells to the dual antigen-positive breast cancer cell line MDA-MB-231 (Fig. 5c). We observed substantial induction of IL-2 secretion and lytic activity of T lymphocytes in the presence of the hemibody pair or the BiTE control in vitro, while individual hemibodies stimulated T cells only at very high concentrations of the constructs to some extent.

To model metastatic disease, immune-deficient mice were challenged i.v. with luciferase-tagged, EGFR and Her2/neu double antigen-positive breast cancer cell line MDA-MB-231. After engraftment of the tumor cells, freshly isolated and non-stimulated PBMCs from a healthy donor were injected i.v., followed by a saline control, the paired EGFR, and Her2/neu specific hemibodies or an EGFR-specific BiTE control, which were administered s.c. (Fig. 5c). In this experiment, the hemibodies were mixed and injected s.c. at one site in order to test whether the constructs might aggregate and thus lose function. However, we found mice treated with EGFR-specific BiTEs or the hemibody combination had rejected all established metastases at day 8 while mice receiving the saline control suffered from progressive disease.

## Discussion

Here we report a T cell-recruiting composite drug, designed to reconstitute and become active exclusively on target.

We presume that the conditional reconstitution of a hemibody pair hinges on at least two major conditions. Firstly, the target antigens must be localized in appropriate proximity to each other on the cell membrane. This might not be the case for combinations of target antigens that are restricted to different membrane areas or where the involved epitopes are positioned too closely or too distantly from each other. In addition, the preconditions regarding density, mobility, and size of the target antigens as well as the anticipated ability of the rather small hemibodies to penetrate into solid tumor parenchyma have yet to be defined. Secondly, the strength of the affinity with which the hemibody pair interacts might be critical. For the constructs investigated here, we observed low-affinity interactions. The addition of further association domains in order to increase affinity and to facilitate VH/VL- pairing may thus cause the hemibodies to dimerize independently from their anchoring antigens, thus jeopardizing the conditional nature of this approach.

The combinatorial nature of the hemibody technique opens up a new dimension of specificity. It may allow selective elimination of human cancer types not amenable to current immunotherapies and differs substantially from other dual or triple antigen-specific strategies designed to enhance avidity to the target. These techniques, however, unleash their cytotoxic potential already after binding to any of the addressed antigens and may thus be associated with substantial on-target/off-tumor toxicity, since most targeted antigens are expressed on healthy and transformed tissues alike^27–29^. Importantly, the antigens addressed by the hemibodies need not to be tumor-specific nor tumor-associated, but unique to cancer in this very combination.

A hemibody pair with its split anti-CD3 variable fragments reconstitutes and is active at the surface of a dual antigen-positive target cell. By discriminating dual-positive targets from single-positive bystanders on single cell level, damage of non-transformed tissues should substantially be impeded, a potential weakness of dual-sensing antibody- or CAR-T cell techniques^30–33^. However, the therapeutic window of this approach, and thus the strict requirement for dual antigen expression, may be narrowed by high-level antigen expression, pre-activation of the effector T cell population populations, and reduced immune checkpoint controls. In addition, further optimizations of the hemibody-architecture may be crucial to extend serum half-lives, stability, and producibility of the constructs and to reduce possible aggregate formation.

It remains to be determined whether hemibodies will induce cytokine release syndromes, a major drawback of BiTEs^11^ or CAR-T cells^34^ directed against antigens such as CD19. In this context, it might be reasonable to address even single target molecules with hemibodies in order to restrict T cell activation exclusively to the tumor site while reducing intravascular T cell activation and systemic cytokine secretion.

Taken together, we envisage that the paired hemibody technique will become a generally applicable platform for combinatorial high precision immunotargeting for the elimination of malignant cells and beyond.

## Methods

### Human cells

Peripheral blood mononuclear cells (PBMC) were obtained by density-gradient centrifugation and red blood cell lysis from peripheral blood of healthy donors in agreement with institutional consent and collection guidelines (Institute for Transfusion Medicine and Haemotherapy, University Hospital Wurzburg and Wurzburg University Ethic Committee, No. 141/17-sc). The human cell lines THP-1 (acute myeloid leukemia, ATCC, No. TIB-202 and DSMZ, No. ACC-16), HT1080 (sarcoma, ATCC, No. CCL-121), U266 (multiple myeloma, ATCC, No. TIB-196), Jurkat (T cell leukemia, DSMZ, No. ACC-282), KMS-12-BM (multiple myeloma, DSMZ, No. ACC-551), Raji (Burkitt lymphoma, ATCC, No. CCL-86), and MDA-MB-231 (ATCC, No. HTB-26) were grown in advanced RPMI-1640 or advanced DMEM supplemented with 200 μM l-glutamine, 10% FBS, penicillin (200 U/mL), and streptomycin (200 μg/mL) (Thermo Fisher Scientific, MA, USA). We routinely tested cell lines for absence of mycoplasma infection. Authentication of the cells was performed by the provider.

### Hemibody and BiTE constructs

DNA coding for hemibodies and bispecific BiTEs were synthesized by GeneArt™ (Thermo Fisher Scientific Inc., Regensburg, Germany) using the scFv sequence derived from a human antibody specific for HLA-A2 (clone 3PF12, GenBank accession AF163308 for anti-HLA-A2/A28 VL and GenBank accession AF163307 for anti-HLA-A2/A28 VH, kindly provided by Nicholas A. Watkins, University of Cambridge, UK)^35^, or published sequences encoding scFv addressing CD3ε (diL2K, the de-immunized version of the mouse monoclonal antibody L2K, Micromet/Amgen)^20^, CD45 derived from the murine IgG1 antibody BC8 (ref. ^36^), Her2/neu (sequence deduced from Herceptin, compound card number CHEMBL1201585, Roche), and EGFR (Sequence deduced from Panitumumab, ATC-Code Lo1XC08, DrugBank DB01269, Amgen). For enhanced expression of the constructs in *E. coli*, an Fh8-tag for cytoplasmic or a pelB-leader for periplasmic expression were fused to the N-terminal end^37^. For purification, a 6- to 8-histidine-tag was placed at the C-terminal end, as well as a FLAG- and/or a myc-tag for detection by ELISA (Supplementary Fig. 1). Sequence data for all engineered hemibodies and BiTE constructs have been deposited at GenBank and are available under GenBank accession numbers MN432899 for VLαCD3(diL2K)-scFvαEGFR, MN432900 for VHαCD3-scFvαHer2, MN432901 for scFvαCD3-scFvαEGFR, MN432902 for VLαCD3-scFvαHLAA2, MN432903 for scFvαCD3-scFvαHLAA2, MN432904 for VHαCD3-scFvαCD45, and MN432905 for scFvαCD3-scFvαCD45.

### Immobilized metal affinity chromatography

*E. coli* lysate was loaded onto a 5 ml HiTrapTALON crude column (GE Healthcare©) using the ÄKTA start chromatography system (GE Healthcare Bio-Sciences, PA, USA). Impurities and endotoxin were removed with five column volumes (CV) IMAC (immobilized metal affinity chromatography) wash buffer (50 mM Na-phosphate pH 7.5, 300 mM NaCl, 10 mM Imidazole pH 8.0), 50 CV IMAC endotoxin removal buffer (50 mM Na-phosphate pH 7.5, 300 mM NaCl, 5 mM Imidazole pH 8.0, 0.2% Triton X-114), and 10 CV IMAC wash buffer at 5 ml/min. Bound protein was eluted with 5 CV IMAC elution buffer (50 mM Na-phosphate pH 7.5, 300 mM NaCl, 150 mM Imidazole pH 8.0) at 3 ml/min. Cell disruption and IMAC purification were performed at 2–8 °C.

### Anion exchange chromatography

The IMAC eluates were further purified by MonoQ anion exchange chromatography using a 1 ml HiTrap Q FF column (GE Healthcare©) after buffer exchange to anion exchange chromatography (AIEX)-binding buffer (50 mM Na-phosphate pH 7.5, 75 mM NaCl) using a HiPrep 26/10 desalting column (GE Healthcare©).

### Size-exclusion chromatography

The eluted proteins were polished through SEC on a HiLoad 16/600 Superdex 200 pg column (GE Healthcare©) with 50 mM Na-phosphate pH 7.5, 300 mM NaCl buffer as eluent at a flow rate of 1 ml/min. To determine the apparent molecular mass of the SEC-purified constructs, the Superdex column was calibrated with cytochrome *C* (12.4 kDa), carbonic anhydrase (29 kDa), albumin (66 kDa), alcohol dehydrogenase (150 kDa), and beta-amylase (200 kDa) as standard proteins (Sigma Aldrich©). AIEX and SEC purification were performed at room temperature using the ÄKTA pure chromatography system (GE Healthcare Bio-Sciences, PA, USA). Remaining endotoxins were removed by EndoTrap® gravity-flow chromatography columns (Hyglos GmbH, Bernried, Germany). Hemibodies and BiTEs were stored at a concentration of ~1 mg/ml at 4 °C in the absence of any stabilizers.

### *Gaussia prinzeps* Luciferase (GpL) fusion proteins

Secreted Flag-tag containing GpL fusion proteins were produced by electroporating (250 V, 1800 µF, maximum resistance, Easyject Plus electroporator; PeqLab, Erlangen, Germany) Hek293 cells (40 × 10^6^ cells in 1 ml medium) with 40 µg of the corresponding expression plasmid (mammalian expression plasmid pCR3). Electroporated cells were recovered overnight in cell culture medium containing 10% FCS. Medium, including dead/non-adherent cells, was changed with fresh 2% FCS-containing medium and cells were then cultivated for an additional 5–7 days. Supernatants were finally collected and cellular debris was removed by centrifugation and filtration (0.2 µm). The integrity of GpL fusion proteins was controlled by anti-Flag-western blotting and concentrations were determined by assessing luciferase activity.

### Equilibrium binding studies

To determine *K*_D_ values of scFvαCD45-GpL, scFvαHLA-A2-GpL, and scFvαCD3-GpL, ~10^6^ Jurkat (scFvαCD45-GpL, scFvαCD3-GpL) or U266 cells (scFvαHLA-A2-GpL) were treated for 1 h at 37 °C with increasing concentrations of these reagents. After removal of unbound scFv-GpL molecules, by washing cells five times with 1 ml ice-cold PBS in Eppendorf reaction tubes (centrifugation 14,000 r.p.m., 1 min), cells were resuspended in 50 µl medium and cell-bound GpL activity was measured using the *Gaussia* Luciferase Assay Kit to determine total binding (New England Biolabs GmbH, Frankfurt a. M., Germany). To obtain corresponding non-specific binding values for scFvαHLA-A2-GpL and scFvαCD3-GpL, HLA-A2-negative KMS-12-BM and CD3-negative U266 cells were similarly treated with these reagents. To determine non-specific binding of scFvαCD45-GpL to Jurkat, cells were incubated with an irrelevant scFv-GpL fusion protein. The differences of the total binding values and the corresponding non-specific binding values were considered as specific binding and were fitted by nonlinear regression using GraphPad Prism 5.0 (GraphPad Software, Inc.).

### Heterologous competition assays

In all, ~10^6^ Jurkat cells were incubated for 1 h at 37 °C with mixtures of 2 or 4 nM scFv-GpL fusion proteins and increasing amounts of the hemibodies or BiTEs as heterologous competitors. Cells were washed five times with ice-cold PBS to remove unbound scFv-GpL molecules. Cell-bound molecules were quantified by assessing GpL activity to determine the IC_50_ values for binding of the scFv-GpL fusion proteins. Finally, the *K*_*i*_-values of hemibodies and BiTEs, which correspond to their *K*_D_-values for the targeted cell surface antigens, were calculated using the above determined *K*_D_-values of scFv-GpL fusion proteins and IC_50_ values.

### Cytokine-ELISA

In all, ~10^5^ tumor cells were co-incubated with 5 × 10^5^ PBMCs from HLA-A2 negative healthy individuals in 96-well plates (tumor to T cell ratio 1:5) in the presence of constructs as indicated. For epitope blocking experiments, tumor cells were pre-incubated for 2 h with epitope-specific scFv blocking constructs at 37 °C prior to adding PBMCs and HLA-A2 and CD45 specific hemibodies at 6 nM. After 12 h, 50–100 µl supernatant was assessed for IL-2 or IFN-gamma secretion by commercially available ELISA Kits (IL-2 ELISA Kit: ABIN1446208, antikoerper-online.de, Aachen, Germany; IFN-gamma ELISA-KIT: hIFNg-EIA-5, MabTag, Friesoythe, Germany, and Quantikine® Human IL-2 ELISA kit: R&D Systems, Minneapolis, MN, USA) according to the manufacturer’s instructions. In some experiments, activity of CD45-specific hemibodies on MCF-7 breast cancer or L-363 human plasma cell leukemia cells was detected. Both cell lines stain negative for CD45 by flow cytometry, but minimal/residual CD45 expression could not be excluded. These data were not considered for further analyses.

### Luciferase-based killing assay

In all, ~10^5^ tumor cells were co-incubated with PBMCs from HLA-A2-negative healthy individuals in 96-well plates (Costar®, Corning Inc., USA) and incubated for 20 h using standard cell culture conditions (37 °C, 5% CO_2_) in the presence of constructs as indicated. Intracellular luciferase activity was monitored to determine killing of the firefly luciferase-expressing THP-1 tumor cells in the presence of HLA-A2 negative PBMCs and constructs. d-Luciferin (Biosynth Inc., USA) was added to a final concentration of 0.5 mM and incubated at 37 °C for 20–30 min. Subsequently, light emission was quantified with an infinite M200 pro ELISA reader (Tecan Ltd, Switzerland). Ten percent DMSO was used to assess maximal killing, which was set at 100%.

### Surface plasmon resonance

Experiments were conducted by GenScript on a Biacore T200. Briefly, a Series S Sensor Chip CM5 was coated with V_L_αCD3-scFvαHLA-A2 using amine coupling methods. Hemibody V_H_αCD3-scFvαCD45 was used as analyte at 100, 200, 400(×2), 800, 1600 (nM) in running buffer (10 mM HEPES, 150 mM NaCl, 3 mM EDTA, 0.005% Tween-20, pH 7.4 at 25 °C). Association contact time was 300 s, dissociation contact time 3600 s at a flow rate of 30 μl/min. Regeneration buffer was 10 mM glycine-HCl (pH 2.0), 10 mM glycine-HCl (pH 1.5). Data were analyzed using the Biacore T200 evaluation software, version 1.0.

### FCS experiments

V_L_αCD3-scFvαHLA-A2 was labeled with a threefold molar excess of the fluorophore Atto655 (AttoTec) by incubation for 40 min at 25 °C in phosphate-buffered saline (PBS) solution, containing 50 mM sodium hydrogen carbonate. Labeled V_L_αCD3-scFvαHLA-A2 was purified by SEC (Sephadex G-25 resin, GE Healthcare). FCS experiments were performed on a confocal fluorescence microscope consisting of an inverse microscope body (Zeiss Axiovert 100 TV) equipped with a diode laser emitting at 637 nm as excitation source (Coherent Cube). Laser light was led into an oil-immersion objective lens (Zeiss Plan Apochromat, ×63, NA 1.4) via a dichroic beam splitter (Omega Optics 645DLRP). The average laser power was adjusted to 400 µW before entering the back aperture of the microscope using an optical density filter. The fluorescence signal was collected by the same objective, filtered by a band-pass filter (Omega Optics 675RDF50), and imaged onto the active area of two fiber-coupled avalanche photodiode detectors (APDs; Perkin Elmer, SPCM-AQRH-15-FC) using a cubic non-polarizing beam splitter (Linos) and multi-mode optical fibers of 100 µm diameter. The signals of the APDs were recorded using a digital hard-ware correlator device (ALV 5000/60 × 0 multiple tau digital real correlator). Atto655-labeled V_L_αCD3-scFvαHLA-A2 was diluted to 1 nM in PBS, pH 7.4, containing 0.3 mg/ml bovine serum albumin (BSA) and 0.05% Tween-20 used as additives to suppress glass-surface interactions. Samples were incubated for 10 min with HLA-A2 antigens at various concentrations, filtered using a 0.2 µm syringe filter, transferred onto a microscope slide, and covered using a coverslip. Sample temperature was adjusted to 25 °C using a custom-built objective heater. For each sample, three individual autocorrelation functions were recorded for 10 min. Recorded autocorrelation functions *G*(*τ*) were fitted using an analytical model for translational diffusion plus an exponential decay that describes additional fluorescence fluctuations of minor amplitude that were faster than diffusion and which arose from intramolecular quenching of the Atto655 conjugate:

1 $$G\left( \tau \right) = \frac{1}{{N\left( {1 + \frac{\tau }{{\tau _{\mathrm{D}}}}} \right)}}\left( {1 + a_1{\mathrm{exp}}\left( { - \frac{\tau }{{\tau _1}}} \right)} \right)$$

*τ* is the lag time, *N* is the average number of molecules in the detection focus, *τ*_D_ is the diffusion time constant, *a*_1_ and *τ*_1_ are amplitude and time constant of the additional relaxation. Application of a model for diffusion in two dimensions was sufficiently accurate because the horizontal dimensions (*x*, *y*) of the detection focus were much smaller than the lateral dimension (*z*) in the applied setup.

### Caspase-3 assay

Staining was performed after co-incubating target cells (THP-1 and monocytes) and T cells (target:T cell ratio = 2:1) with or without the hemibodies and BiTE constructs directed against CD45 or HLA-A2 respectively for 4 h as indicated. For analysis, surface staining for HLA-A2 was performed first, using anti-HLA-A2-APC (Biolegend, mouse IgG2b, used at 1 μl in 50 μl), followed by fixation and permeabilization of the cells (Fix + Perm, BD Biosciences). Rabbit anti-active Caspase-3-PE (BD Biosciences, clone C92-605, 3 μl in 50 μl) was subsequently added for 30 min. Cells were washed with 1× PBS + 5% human serum (HS, PAA Laboratories) and analyzed on a BD-FACS Canto-II. Stepwise gating was performed by first using a FSC/SSC live gate followed by focusing on different target populations (FCS/HLA-A2 gate), thus evaluating HLA-A2-positive and HLA-A2-negative targets separately for active caspase-3 positivity. Various conditions (titrated hemibody concentrations, various HLA-A2 positive and negative tumor cell lines) were tested in at least 12 independent experiments (including THP-1 cells (9×) and monocytes as bystanders (2×).

### In vivo mouse models

All animal experiments received approval by the appropriate authorities (Regierung von Unterfranken, No. RUF-55.2-2531.01-79/11) and comply with all relevant ethical regulations for animal testing and research.

For the in vivo model shown in Fig. 4, immune-deficient NOD scid *Il2rg*^−/−^ (NSG mice, stock number 5557) and BalB/c mice (stock number 028) were purchased from The Jackson Laboratory (Bar Harbor, Maine, USA) and maintained in a certified animal facility (ZEMM, Center for Experimental Molecular Medicine, Würzburg) in accordance with European guidelines. 1 × 10^6^ luciferase-positive THP-1 tumor cells and unstimulated HLA-A2 negative donor T lymphocytes (1 × 10^6^ CD4- and 7.6 × 10^4^ CD8-positive memory T cells) were injected intravenously into the tail veins of 6–12-week-old female mice at day 1. Memory T cells were isolated from healthy donors using magnetic beads (Miltenyi Biotec GmbH, Bergisch Gladbach, Germany). Because of their limited life-span in NSG mice, a fraction of these T cells was expanded with CD3/CD28 CTS paramagnetic beads (Dynabeads) and IL-2 and injected on days 22 and 28 after transplantation (5 × 10^6^ CD4 and 3.3 × 10^6^ CD8 memory T cells on day 22, and 2.8 × 10^6^ CD4 and 2.2 × 10^6^ CD8 memory T cells on day 28) according to established protocols^38^. After successful engraftment of tumor cells, constructs were diluted in sterile PBS and applied subcutaneously at distant sites (nuchal fold and abdominal skin fold). Two micrograms construct was applied daily from day 7 until day 14 and 8 µg from day 14 to 39 per mouse. Tumor bearing animals were sacrificed when they reached criteria for euthanasia. To monitor growth of luciferase-positive tumor cells weekly, each mouse received i.p. 220 µl anesthesia cocktail (Ketavet 8 mg/ml and Xylavet 1.6 mg/ml) and 200 µl Luciferin (30 mg/ml). Luciferase activity was assessed using an IVIS Lumina XR Real-Time Bioluminescence Imaging System. Considerations on sample size for statistical significance are provided in Supplementary Table 1.

For the in vivo model shown in Fig. 5b, immune-deficient NOD scid *Il2rg*^−/−^ were challenged with 6 × 10^5^ RAJI and THP-1 tumor cells s.c. as indicated. 12 × 10^6^ unstimulated memory T cells from a healthy HLA-A2-negative donor were isolated by magnetic beads (Miltenyi Biotec GmbH, Bergisch Gladbach, Germany) and injected i.v. (E:T = 20:1). After engraftment, saline, the combination of HLA-A2 and CD45-specific hemibodies or BiTEs (8 µg each) against HLA-A2 were applied s.c. once daily for 7 days at distant sites. Luciferase activity was assessed using an IVIS Lumina XR Real-Time Bioluminescence Imaging System on days 1 and 8.

For the in vivo model shown in Fig. 5c, the experiment was carried out at Charles River Discovery Research Services Germany GmbH (Freiburg, Germany). 2 × 10^6^ luciferase-expressing MDA-MB-231 tumor cells were injected into the tail vein of immune-deficient NOD-Prkdc^em26Cd52^ Il2rg^em26Dc22^/NjuCrl (NCG) mice (Charles River). To monitor tumor cell engraftment, in vivo luminescence imaging of sedated animals was carried out 2 days after tumor cell injection ( = day 0 of experiment). Animals were selected for assignment to treatment groups based on comparable signal intensity. The next day ( = day 1), 1 × 10^7^ human PBMCs from a healthy donor were injected i.v. into all animals (E:T = 5:1). This was directly followed by s.c. application of either saline (negative control), the combination of V_L_αCD3-scFvαEGFR and V_H_αCD3-scFvαHer2/neu hemibodies or the scFvαCD3-FvαEGFR BiTE control. Each animal received 8 µg of the indicated constructs per day for 7 days and mice were subjected to in vivo luminescence imaging on day 8.

### Reporting summary

Further information on research design is available in the Nature Research Reporting Summary linked to this article.

## Supplementary information

Supplementary Information

Reporting Summary

## Data Availability

Data presented in this study are available in the Article, Supplementary Information or available from the corresponding author upon reasonable request. Sequence data for all engineered hemibodies and BiTE constructs have been deposited at GenBank and are available under GenBank accession numbers MN432899 for VLαCD3(diL2K)-scFvαEGFR, MN432900 for VHαCD3-scFvαHer2, MN432901 for scFvαCD3-scFvαEGFR, MN432902 for VLαCD3-scFvαHLAA2, MN432903 for scFvαCD3-scFvαHLAA2, MN432904 for VHαCD3-scFvαCD45, and MN432905 for scFvαCD3-scFvαCD45. A reporting summary for this article is available as a Supplementary Information file.
