# Supplementary material for: On-target restoration of a split T cell-engaging antibody for precision immunotherapy
Source: Nat Commun. 2019 Nov 26;10:5387. doi: 10.1038/s41467-019-13196-0 (PMC6879491; doi:10.1038/s41467-019-13196-0)
Supplement: Supplementary file 1 — Supplementary Information [file 41467_2019_13196_MOESM1_ESM.pdf]

Supplementary Information:

a

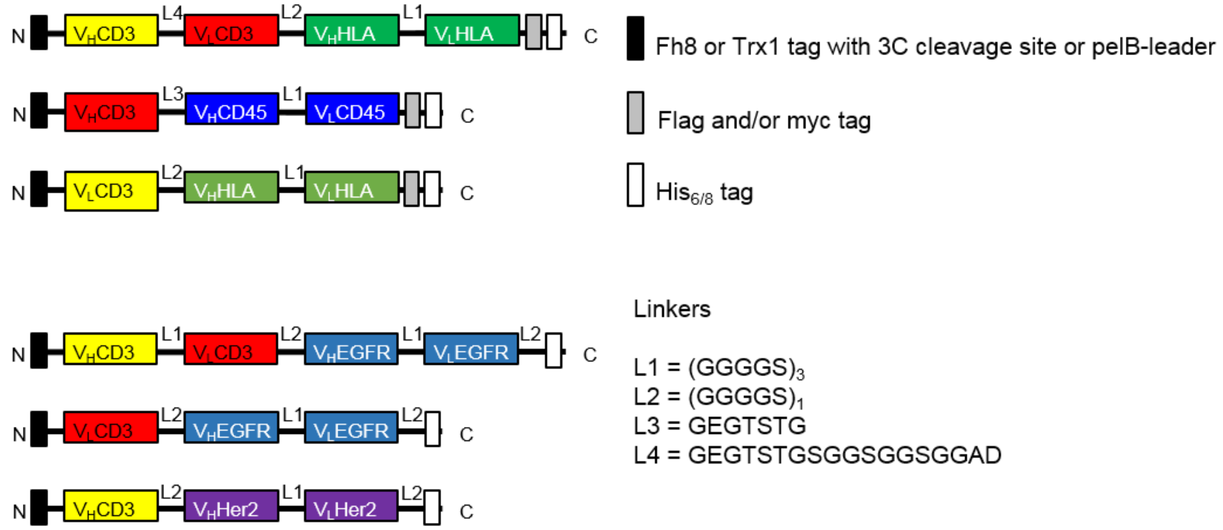

b

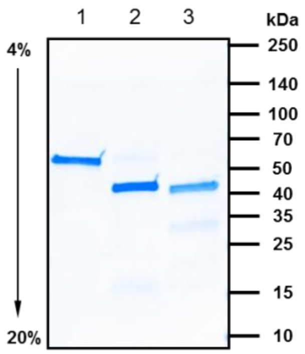

c

construct	Total amount [mg]/10l	Purity [%]
scFvαCD3-scFvαHLA-A2	2.6	93
V_HαCD3-scFvαCD45	2.5	86
V_LαCD3-scFvαHLA-A2	1.5	64

Supplementary Figure 1. **(a)** Schematic representation of the constructs used in this study. **(b)** SDS-PAGE analysis of hemibody constructs. 1 µg of scFvαCD3-scFvαHLA-A2 (1), V_HαCD3-scFvαCD45 (2) and V_LαCD3-scFvαHLA-A2 (3) SEC-purified construct was subjected to gradient SDS-PAGE under reducing conditions followed by Coomassie staining, a typical profile is shown

(c) Table summarizing yields of SEC-purified constructs. Protein amounts are from *E. coli* broth with an average OD_{600} of 5.0.

Supplementary Figure 2: Long term stability.

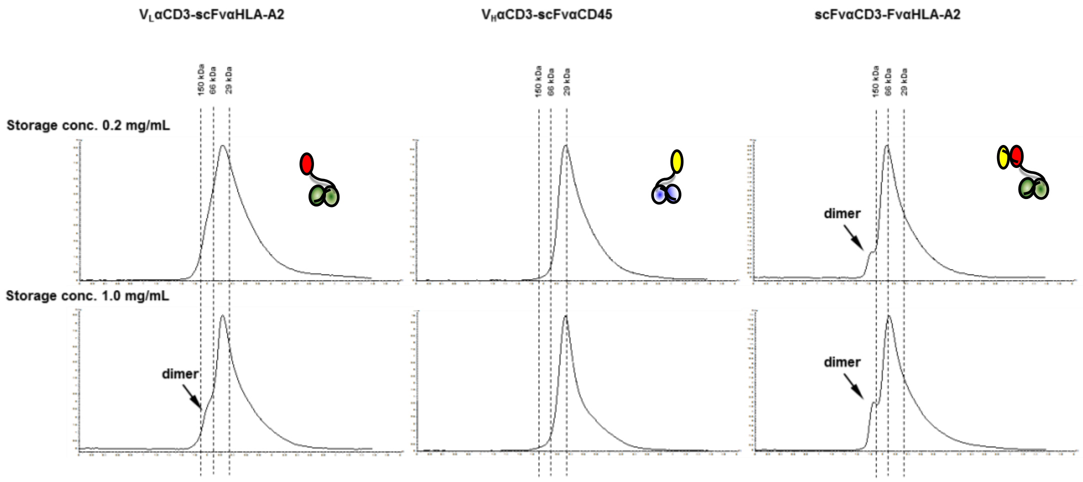

SEC purified constructs were stored at the given concentrations over a period of one month at 2-8°C in diluent buffer. Subsequently, samples were analyzed by size exclusion chromatography (SEC). SEC conditions: Sample volume loaded on column = 50 µL, Column = Superdex200 Increase 5/150 GL, running buffer = diluent buffer, flow rate = 0.4 mL/min, temperature = RT, diluent buffer = 50mM Na phopshate pH7.5, 300 mM NaCl, typical profiles are shown.

Supplementary Figure 3

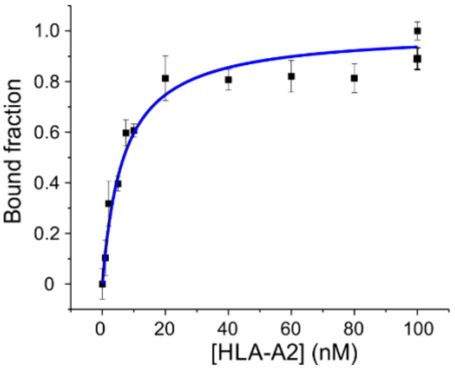

Binding isotherm of V_LαCD3-scFvαHLA-A2/HLA-A2 interaction: : Diffusion time constants measured by FCS of 1nM V_LαCD3-scFvαHLA-A2 in presence of increasing concentration of

HLA-A2 antigens, normalized to the fraction of bound V_LαCD3-scFvαHLA-A2. The blue line is a fit to the data using the conventional model for a bi-molecular binding isotherm.

Supplementary Table 1: Estimated group size for the NSG mouse model.

Group size for experiments in the NSG mouse model:						
Mice per group	5	6	7	8	9	10
Effectsize[1]	2.0	1.8	1.6	1.5	1.4	1.3
SD fraction[2]	0.49	0.56	0.61	0.66	0.71	0.75

[1]mean difference in standard deviation (SD) units detectable with 80% power at 2-sided 0.05 level of significance with 2-sample t-test;
[2]SD as a fraction of biologically meaningful difference (inverse of effect size).

The sample size to achieve statistically significant data for this model was calculated based on an estimated effect size of 2 according to Ms. Katherine Guthrie, Fred Hutchinson Cancer Research Center, Seattle, USA, using GPOWER 3.1.2 [1].

References for Supplementary Figures and Material

1. Erdfelder, E., Faul, F., & Buchner, A. GPOWER: A general power analysis program. *Behavior Research Methods, Instruments, & Computers* **28**, 1-11. (1996).
