# Supplementary material for: On-target restoration of a split T cell-engaging antibody for precision immunotherapy
Source: Nat Commun. 2019 Nov 26;10:5387. doi: 10.1038/s41467-019-13196-0 (PMC6879491; doi:10.1038/s41467-019-13196-0)
Supplement: Supplementary file 2 — Reporting Summary [file 41467_2019_13196_MOESM2_ESM.pdf]

nature research

Corresponding author(s): Gernot Stuhler

Last updated by author(s): Aug 28, 2019

Reporting Summary

Nature Research wishes to improve the reproducibility of the work that we publish. This form provides structure for consistency and transparency in reporting. For further information on Nature Research policies, see Authors & Referees and the Editorial Policy Checklist .

Statistics

For all statistical analyses, confirm that the following items are present in the figure legend, table legend, main text, or Methods section.

n/a	Confirmed	
[ ]	[x]	The exact sample size (*n*) for each experimental group/condition, given as a discrete number and unit of measurement
[ ]	[x]	A statement on whether measurements were taken from distinct samples or whether the same sample was measured repeatedly
[ ]	[x]	The statistical test(s) used AND whether they are one- or two-sided<br>*Only common tests should be described solely by name; describe more complex techniques in the Methods section.*
[x]	[ ]	A description of all covariates tested
[x]	[ ]	A description of any assumptions or corrections, such as tests of normality and adjustment for multiple comparisons
[x]	[ ]	A full description of the statistical parameters including central tendency (e.g. means) or other basic estimates (e.g. regression coefficient) AND variation (e.g. standard deviation) or associated estimates of uncertainty (e.g. confidence intervals)
[x]	[ ]	For null hypothesis testing, the test statistic (e.g. *F*, *t*, *r*) with confidence intervals, effect sizes, degrees of freedom and *P* value noted<br>*Give P values as exact values whenever suitable.*
[x]	[ ]	For Bayesian analysis, information on the choice of priors and Markov chain Monte Carlo settings
[x]	[ ]	For hierarchical and complex designs, identification of the appropriate level for tests and full reporting of outcomes
[ ]	[x]	Estimates of effect sizes (e.g. Cohen's *d*, Pearson's *r*), indicating how they were calculated

Our web collection on statistics for biologists contains articles on many of the points above.

Software and code

Policy information about availability of computer code

Data collection	Mouse experiments: Living Image Software 4.5.5, In vitro Assays: ImageLa, Tecan SPARKCONTROL Method Editor V2.2 , Unicorn V 6.3
Data analysis	Mouse experiments: Living Image Software 4.5.5, , In vitro Assays: FlowJo V 8.8.6/V10, ImageLab, GraphPad Prism V 7, Unicorn V 6.3

For manuscripts utilizing custom algorithms or software that are central to the research but not yet described in published literature, software must be made available to editors/reviewers. We strongly encourage code deposition in a community repository (e.g. GitHub). See the Nature Research guidelines for submitting code & software for further information.

Data

Policy information about availability of data

All manuscripts must include a data availability statement. This statement should provide the following information, where applicable:

- Accession codes, unique identifiers, or web links for publicly available datasets
- A list of figures that have associated raw data
- A description of any restrictions on data availability

Sequence data from all engineered constructs are submitted to GenBank. The datasets are available from the corresponding author upon reasonable request.

Field-specific reporting

Please select the one below that is the best fit for your research. If you are not sure, read the appropriate sections before making your selection.

[x] Life sciences [ ] Behavioural & social sciences [ ] Ecological, evolutionary & environmental sciences

For a reference copy of the document with all sections, see nature.com/documents/nr-reporting-summary-flat.pdf

Life sciences study design

All studies must disclose on these points even when the disclosure is negative.

Sample size	2 to 6 female mice of same age per group as indicated in respective figures
Data exclusions	No relevant data was excluded
Replication	Functional assays were performed in dublicates or triplicates. Flow cytometry analyses were derived from healthy volunteers as indicated.
Randomization	Allocation of samples/animals into experimatal groups was random
Blinding	blinding was not possible to ensure correct treatment and grouping.

Reporting for specific materials, systems and methods

We require information from authors about some types of materials, experimental systems and methods used in many studies. Here, indicate whether each material, system or method listed is relevant to your study. If you are not sure if a list item applies to your research, read the appropriate section before selecting a response.

Materials & experimental systems

n/a	Involved in the study	
☐	☒	Antibodies
☐	☒	Eukaryotic cell lines
☒	☐	Palaeontology
☐	☒	Animals and other organisms
☒	☐	Human research participants
☒	☐	Clinical data

Methods

n/a	Involved in the study	
☒	☐	ChIP-seq
☐	☒	Flow cytometry
☒	☐	MRI-based neuroimaging

Antibodies

Antibodies used	anti-HLA-A2-APC (Biolegend, mouse IgG2b) , Rabbit anti-active Caspase-3-PE (BD Biosciences, clone C92-605).
Validation	antibodies have been validated by manufacurer

Eukaryotic cell lines

Policy information about cell lines

Cell line source(s)	Jurkat, U266, KMS-12-BM, THP-1, Raji, HT1080, MDA-MB 231: DMSZ and ATCC as reported in M&M.
Authentication	Authentication by provider
Mycoplasma contamination	All cell lines were tested negative for mycoplasma contamination.
Commonly misidentified lines (See ICLAC register)	-

Animals and other organisms

Policy information about studies involving animals; ARRIVE guidelines recommended for reporting animal research

Laboratory animals	Immune deficient NOD scid Il2rg-/- (NSG mice, stock number 5557), female<br>BalB/c mice (stock number 028), female<br>Immune deficient NOD-Prkdcem26Cd52 Il2rgem26Dc22/NjuCrl (NCG) mice, female<br>All mice were purchased from The Jackson Laboratory (Bar Harbor, Maine, USA) and maintained in a certified animal facility (ZEMM, center for experimental molecular medicine, Würzburg) in accordance with European guidelines.
Wild animals	No wild animals were involved in this study.
Field-collected samples	This study did not involve field-collected samples.

Ethics oversight	Animal experiments have been approved by the appropriate authorities and performed at the ZEMM, center for experimental molecular medicine, Würzburg (experiment number: 2-2544-24) or Charles River Discovery Research Services Germany GmbH (Freiburg, Germany)

Note that full information on the approval of the study protocol must also be provided in the manuscript.

Flow Cytometry

Plots

Confirm that:

- [x] The axis labels state the marker and fluorochrome used (e.g. CD4-FITC).
- [x] The axis scales are clearly visible. Include numbers along axes only for bottom left plot of group (a 'group' is an analysis of identical markers).
- [ ] All plots are contour plots with outliers or pseudocolor plots.
- [x] A numerical value for number of cells or percentage (with statistics) is provided.

Methodology

Sample preparation	PBMC from HLA-A2 negative healthy donors were mixed for 4h with tumor cell lines and repective antibody constructs, followed by surface staining and intracellular caspase-3 staining.
Instrument	BD-FACS Canto-II
Software	FlowJo (Treestar)
Cell population abundance	n.a.
Gating strategy	Cell were gated according to light scatter and HLA-A2 expression (as shown in the Figure).

- [x] Tick this box to confirm that a figure exemplifying the gating strategy is provided in the Supplementary Information.
